# Determinants of ADL and IADL disability in older adults in southeastern Poland

**DOI:** 10.1186/s12877-019-1319-4

**Published:** 2019-10-31

**Authors:** Agnieszka Ćwirlej-Sozańska, Agnieszka Wiśniowska-Szurlej, Anna Wilmowska-Pietruszyńska, Bernard Sozański

**Affiliations:** 1 Department of Medicine, Institute of Physiotherapy, University of Rzeszow, Rzeszow, Poland; 2 Department of Medicine, Lazarski University, Warsaw, Poland; 3 Center for Innovative Research in Medical and Natural Sciences, University of Rzeszow, Rzeszow, Poland

**Keywords:** Aged, Disability, Factors, Basic activities of daily living, Instrumental activities of daily living

## Abstract

**Background:**

The extension of the life span has led to an increase in the number of older people and an increase in the prevalence of disability in people over 60 years of age. The aim of this study was to assess the prevalence of ADL and IADL disability and to analyze its determinants among people aged 60 and older living in southeastern Poland.

**Methods:**

This cross-sectional study was carried out among a randomly selected, representative population of people aged 60 and older living in southeastern Poland. Disability was assessed using the Katz Index of Independence in Basic Activities of Daily Living and Instrumental Activities of Daily Living. Logistic regression models were used to identify the factors related to ADLs and IADLs. For the variables that were included in the above models, their clustered influence on the increase in the odds ratio for the occurrence of an ADL or IADL limitation was also examined.

**Results:**

The research results show that 35.75% of the participants reported at least one problem with IADLs. At least one problem with ADLs was reported by 17.13% of the participants. The most significant modifiable factors influencing the occurrence of disability were the presence of barriers in the participant’s environment, poor relations with relatives, a lack of social contacts, multimorbidity and pain. A multiple increase in the odds ratio of disability was found with the presence of pairs of analyzed factors. The highest odds ratio of at least one ADL limitation was observed for the combination of barriers in the participant’s environment with multimorbidity (OR 74.07). With regard to IADL disability, the highest odds ratio was observed for the combination of pain on the VAS scale ≥3 points with older age (OR 19.47).

**Conclusions:**

The study showed a high prevalence of ADL and IADL disability in older people living in southeastern Poland. It also indicated the extent to which modifiable factors influenced the occurrence of disability and the extent to which the risk of disability increased with the presence of pairs of factors, especially those that included environmental barriers in the participant’s environment.

## Background

Over the last 30 years, significant changes in the age structure of the inhabitants of Poland have been observed. At the end of 2017, the population of Poland was 38.4 million, of which over 9 million were people aged 60 and over. There are particularly high percentages of older people in Poland in the following age ranges: approximately 30% aged 60–64, 25% aged 65–69 and 18% aged 80 and over [1].

According to a worldwide report on disability, approximately 1 billion people experience disability worldwide [2]. Over 45% of older adults aged 60 and over have difficulty performing everyday activities, and over 250 million people experience disabilities to a moderate or significant degree [3]. According to Eurostat data regarding Poland, over 34% of people aged 60 and over report moderate or significant difficulties in performing everyday activities [4]. Disability is commonly defined as a difficulty in performing activities necessary for independent living, such as basic activities of daily living (ADLs) and complex instrumental activities of daily living (IADLs) [5]. In Europe, the disability rate among older people measured by the presence of at least one ADL disability varies between 11 and 44%, and the rate measured by the presence of at least one IADL disability varies between 8 and 40% and is dependent on age and gender [6–8].

Disability among older people is the result of not only health problems but also the interactions between health condition, activity and participation, personal factors and environmental factors [9]. To unify the assessment of problems and difficulties related to functioning, the World Health Organization (WHO) developed the International Classification of Functioning, Disability and Health (ICF) based on a biopsychosocial model of functioning and disability [10]. The occurrence and level of disability are related to the health conditions and the resulting disabilities in interacting with the physical and social world [11].

Previous studies have shown that the incidence of disability in older people is influenced by factors such as alcohol consumption, smoking, cognitive disorders, chronic diseases, upper and lower limb dysfunctions, high consumption of pharmaceuticals, high or low body mass index (BMI), a lack of physical activity, a poor health self-assessment, a low level of social activity [12] and the presence of environmental barriers [13]. Other risk factors include age, prevalence of pain, stroke, depression and falls [9, 14].

Limitations in functioning and dependence on other people in performing daily activities lead to a worse quality of life for older people and an increase in the social costs of care and health [15]. A comprehensive understanding of the factors that have an impact on daily functioning in the range of performed ADLs and IADLs is very important for planning targeted strategies for the development of social, health care and promotion activities. It is important to conduct research and determine the factors that particularly influence the development of disability in older people. Such research is important because there is high variability in the prevalence of disability in relation to the socioeconomic position of a region, among other factors [16]. Countries with less developed economies and weaker social policies are characterized by higher levels of disability among older people and an earlier onset of such disability [7]. Poland belongs to a group of countries with one of the highest disability rates of older people [8], and the region of southeastern Poland is one of the poorest regions of Poland [17].

Due to the different socioeconomic conditions in Poland than in other European countries, we decided to determine the prevalence of at least one limitation in both ADLs and IADLs in a representative population of people aged 60 and over living in southeastern Poland. Moreover, the odds of having limitations in performing simple and complex daily activities in the study group were assessed regarding particular factors and pairs of factors.

## Methods

### Study design and participants

This cross-sectional study was carried out by researchers at the University of Rzeszow among a randomly selected representative population of people aged 60 and older living in southeastern Poland (region of the Podkarpackie Voivodeship). There are 350,000 people aged 60 and over in Podkarpackie Voivodeship [18]. The inclusion criteria for participation in the study group were an age of 60 or older, normal cognitive state (Abbreviated Mental Test Score (AMTS) > 6 points), and provision of informed consent for participation in the study.

The *operational database* was the PESEL (personal identity number) database created by the Ministry of Interior and Administration. The draw was made by the Voivodeship IT Center and the Regional Data Bank at the Podkarpackie Voivodeship Office in Rzeszow from among all residents living in the Podkarpackie Voivodeship who meet the age criterion, i.e. age range 60 years and more. 34,530 people were drawn, and a random sample of the main research sample of 2350 respondents was drawn from this group. The draw was made using the SPSS program, without replacement of already drawn respondents. A simple sampling method was made, i.e. for all units the probability of random sampling was the same. Due to this method, a representative sample was obtained for this region corresponding to the structure of the population in this age group.

**Fig. 1:**
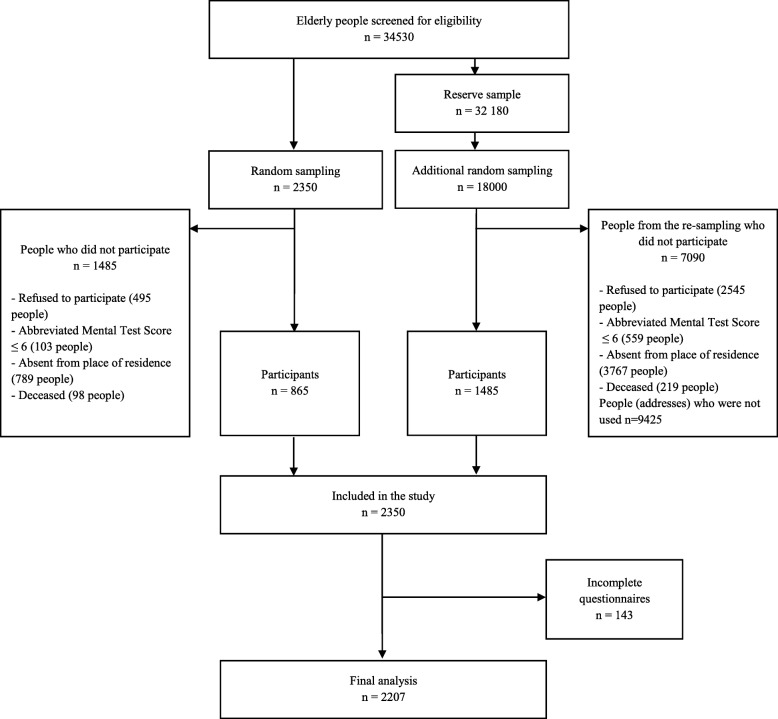
Flow diagram of the study population

The calculation of the sample size was based on the following assumptions: a 95% (0.95) confidence level and a fraction size of 0.5 with a maximum estimation error of 3%. A flow chart shows the participant selection and drop-out process (Fig. 1).

The study was carried out by appropriately prepared and trained interviewers who conducted direct, pen-and-paper interviews at the participants’ places of residence. Households were replaced after 3 unsuccessful interviewer visits of the interviewer due to the resident not being at home or an older person’s refusal to participate in the survey of an older person, death or inability to participate. Eventually, 2207 complete interviews were included in the analysis.

### Data collection

To assess the participants’ cognitive states, an abbreviated version of the AMTS questionnaire [19] was used. Since the interview was conducted directly with older persons, this questionnaire served as a screening criterion (AMTS > 6 points) to ensure the collection of reliable answers.

Information on socioeconomic variables such as age, sex, place of residence, marital status, education, and income was collected by means of an Socioeconomic Status Index.

The Katz Index of Independence in Activities of Daily Living [20] was used to assess basic activities in everyday life, whereas the Lawton Instrumental Activities of Daily Living Scale was used to assess complex daily activities [21].

To examine various factors affecting the functioning of older people in Poland, codes from the WHOICF checklist were selected [10]. A review of the literature and a selection (mapping) of relevant items/questions for the selected categories (category) in accordance with WHO recommendations were carried out [22]. The selected codes were assigned to questions from the standardized questionnaires (e.g., WHODAS 2.0, WHOQOL). Questions regarding the physical activity of older adults were prepared according to the recommendations of the European Network for Action on Aging and Physical Activity [23]. Based on pilot study (that preceded the main study), the prepared tools were checked. The repeatability and correlations among the questionnaires included in the mapping were assessed. To reduce the number of questions that would be asked in the main study, the questions that significantly differentiated the population depending on the functional level in the pilot study were selected.

In the main study, the following information concerning physical health was collected: height and body mass (including BMI calculations and ICF b530 Weight maintenance functions), pain occurrence (ICF b280 Sensation of pain) and the number of chronic diseases (a quantitative variable related to health conditions was selected as a general reference to the ICD-10 classification complementary to the ICF and a popular indicator of multimorbidity and the maintenance of good health in older people) [24]. There were questions about physical activity, including the amount of time devoted to any physical activity during the week (at least moderate physical activity, defined as activity causing at least a slight shortness of breath, sweating, and fatigue), as well as about planned physical exercises performed to improve strength and endurance (ICF d570 Looking after one’s health, including the maintenance of appropriate physical activity) [25]. Furthermore, the researchers also collected information on social activity and participation, including participation in groups or social organizations (ICF d910 Community life), and maintaining good relations with relatives (ICF d760 Family relationships) and acquaintances (ICF d720 Complex interpersonal interactions). The participants were also asked about their living environments, including the existence of barriers and obstacles (including architectural, communication, social and other barriers) (a comprehensive selection of the environmental factors included in the Short List of Environment on the WHO ICF checklist) and their housing conditions (ICF e155 Design, construction and building products and technology of buildings for private use). For the physical health analysis, responses were given in the form of quantitative variables (BMI, the VAS pain scale, and the number of diseases).

To use the collected information in a logistic regression model, the obtained information was coded as dichotomous data (yes/no), except for housing conditions, which had three options for further analysis. The cut-off point for the assessment of physical activity was established based on the WHO recommendations [25].

### Statistical analysis

For the analysis, the participants were divided into persons without any difficulties and persons reporting at least one ADL limitation and at least one IADL limitation. Dichotomous variables were created, with a value of 1 if the participant showed a limitation in one or more ADLs (1+ ADLs) and IADLs (1+ IADLs) and a value of 0 if the participant did not show any limitations. These cut-offs are provided by the SHARE project [7]. The following sections present only the results of people with at least one difficulty in the ADL and IADL scales. The data were analyzed using Statistica version 13.1. Demographic data are presented as descriptive statistics. Two logistic regression models were used to identify factors related to ADLs and IADLs. The level of significance was set at *p* <  0.05. The chi-square test (in the case of qualitative variables) and the Mann-Whitney test (in the case of quantitative variables) were used for the initial analysis of the relationship between the individual demographic variables and ADLs and IADLs. The normal distribution of the quantitative variables was verified using the Shapiro-Wilk test. Logistic regression models were used to identify sets of factors that had statistically significant effects on the occurrence of at least one ADL limitation and at least one IADL limitation in the entire study group. The model parameters were estimated by means of stepwise regression (forward selection). The quality of the model estimation was tested using the Hosmer-Lemeshow test and pseudo R^2^ values. For the variables that were included in the above models, their clustered influence on the increase in the odds ratio for the occurrence of an ADL or IADL limitation was also examined when the factors occurred in pairs for people who did not report these variables. The following dichotomous variables were used for this analysis: age, pain level and the number of diseases. In order to obtain the dichotomous variable, the WHO age division was used, i.e., the 60–74 years (early old age) and 75 and more (late old age and very old age) group [26], while the number of diseases was divided according to the definition of multimorbidity, i.e., 0–1 diseases and 2 and more diseases [27]. The pain level variable as measured by the VAS scale was divided into 0–2 points and 3 and more points. Three points was adopted as the cut-off point because this level significantly differentiated the respondents with at least one ADL limitation and one IADL limitation. The significance level was set at *p* <  0.05.

## Results

### Characteristics of the studied population

In total, the study included 2207 people aged 60 and older, including 1325 women and 882 men. The average age of the participants was 72.12 (SD = 7.77). The overwhelming majority of participants had primary or vocational education (63.43%) and were in a relationship (62.62%). Among the majority of participants who answered the income question, the average income per household member was PLN 2000 or less per month. On average, each participant had an average of 4.94 (SD = 3.55) chronic diseases, while their average pain level as measured by the VAS scale (0–10) was 3.58 points (SD = 2.91). Most of the participants did not perform at least a moderate level of physical activity for 150 min/week (71.05%) and did not perform physical exercises aimed at strengthening the muscles and improving physical fitness (77.07%). Moreover, most of the participants did not belong to a social group (77.75%). On the other hand, the majority of people maintained social contacts (62.62%) and claimed that they had good relations with their relatives (63.25%). Almost half of the surveyed group (48.71%) stated that their environments had some barriers and obstacles (communication, social, or architectural), and only 40.82% of participants were satisfied with their living conditions (Table 1).

**Table 1:** Characteristics of the study population of people aged 60 and more (*n* = 2207)

Variables		Total	Difficulty with ADL		Difficulty with IADL	
		Number (%) Mean (SD)	Number (%) Mean (SD)	*p* value	Number (%) Mean (SD)	*p* value
Socioeconomic						
Age		72.12 (7.77)	77.77 (7.41)	< 0.001^b^	76.43 (7.29)	< 0.001^b^
Gender	Females	1325 (60.04)	254 (19.17)	0.002^c^	509 (38.42)	0.001 ^c^
	Males	882 (39.96)	124 (14.06)		280 (31.75)	
Place of residence	Town	931 (42,18)	122 (13.10)	< 0.001 ^c^	280 (30.08)	< 0.001 ^c^
	Village	1276 (57.82)	256 (20.06)		509 (39.89)	
Marital status	In relationship	1382 (62.62)	173 (12.52)	< 0.001 ^c^	396 (28.65)	< 0.001 ^c^
	Single	825 (37.38)	205 (24.85)		393 (47.64)	
Education	At most vocational	1400 (63.43)	282 (20.14)	< 0.001 ^c^	551 (39.36)	< 0.001 ^c^
	At least secondary	807 (36.57)	96 (11.90)		238 (29.49)	
Income^a^	up to 2000 PLN and less / person	1061 (69.71)	207 (19.51)	0.002 ^c^	414 (39.02)	< 0.001 ^c^
	2001 PLN and more	461 (30.29)	59 (12.80)		130 (28.20)	
Physical health						
BMI		27.46 (4.69)	27.49 (5.28)	0.346 ^b^	27.59 (4.90)	0.512 ^b^
Pain on the VAS scale		3.58 (2.91)	5.73 (2.71)	< 0.001^b^	5.18 (2.80)	< 0.001^b^
Number of chronic diseases		4.94 (3.55)	6.87 (3.58)	< 0.001 ^b^	6.34 (3.70)	< 0.001 ^b^
Physical activity						
Physical activity performed daily, with a minimum of 150 min per week	No	1568 (71.05)	333 (21.09)	< 0.001 ^c^	626 (39.92)	< 0.001 ^c^
	Yes	639 (28.95)	45 (7.04)		163 (25.51)	
Physical exercises performed to strengthen muscles and improve physical performance	No	1701 (77.07)	308 (18.11)	0.025 ^c^	632 (37.15)	0.012 ^c^
	Minimum once a week	506 (22.93)	70 (13.83)		157 (31.03)	
Social activity and participation						
Social activity and participation	No	1716 (77.75)	303 (17.66)	0.217 ^c^	603 (35.14)	0.264 ^c^
	Yes	491 (22.25)	75 (15.27)		186 (37.88)	
Maintenance of social contacts	No	825 (37.38)	253 (30.67)	< 0.001 ^c^	420 (50.91)	< 0.001 ^c^
	Yes	1382 (62.62)	125 (9.04)		369 (26.70)	
Maintenance of good relations with relatives	No	811 (36.75)	252 (31.07)	< 0.001 ^c^	420 (51.79)	< 0.001 ^c^
	Yes	1396 (63.25)	126 (9.03)		369 (26.43)	
Environment						
Presence of barriers and obstacles (including architectural, communication, social and other barriers) in the environment of the respondent	No	1132 (51.29)	38 (3.36)	< 0.001 ^c^	174 (15.37)	< 0.001 ^c^
	Yes	1075 (48.71)	340 (31.63)		615 (57.21)	
Assessment of the residential conditions related to the presence of barriers/facilitators to everyday functioning	Unsatisfied / very unsatisfied	577 (26.14)	119 (20.62)	< 0.001 ^c^	227 (39.34)	< 0.001 ^c^
	Neither satisfied / nor unsatisfied	729 (33.03)	138 (18.93)		282 (38.68)	
	Satisfied / very satisfied	901 (40.82)	121 (13.43)		280 (31.08)	

^a^the lack of data of 685 people

^b^Mann-Whitney test

^c^chi-square test

The research results showed that 35.75% of participants reported at least one problem with IADLs. Most problems with IADLs were found to be related to walking farther than the normal walking distance/moving within the community (27.46%). At least one problem with ADLs was reported by 17.13% of people. Most often, the participants had problems getting out of bed and moving around (17.54%). The prevalence of at least one problem with ADLs and IADLs increased gradually in the older age groups (Table 1).

Problems with ADLs and IADLs occurred significantly more frequently for the following individuals: older people, women, lonely people, those with lower incomes, those with lower physical activity levels, those who did not engage in physical exercise, those who did not maintain social contacts, those with worse relations with their relatives, those living in an environment with barriers and obstacles, those with more chronic diseases and those with higher levels of pain (Table 1).

The most common limitations in ADL in the study group were bathing and showering (8.38%) and dressing (6.52%). In IADL, moving within the community posed the most problems (27.46%). The percentage of people experiencing at least one problem with ADL and IADL increased in older age groups (Table 2).

**Table 2:** Functional disability in ADLs and IADLs of older people (*n* = 2207)

Variables		Number (%)
ADL	Bathing and showering	185 (8.38)
	Dressing	144 (6.52)
	Toilet hygiene (getting to the toilet, cleaning oneself, and getting back up)	129 (5.85)
	Transferring - functional mobility	123 (5.57)
	Self-feeding (not including cooking or chewing and swallowing)	109 (4.94)
	Continence	78 (3.53)
IADL	Moving within the community	606 (27.46)
	Shopping for groceries and necessities	208 (9.42)
	Cleaning and maintaining the house	205 (9.29)
	Managing money	198 (8.97)
	DIY/washing	190 (8.61)
	Preparing meals	137 (6.21)
	Using the telephone or other form of communication	74 (3.35)
	Taking prescribed medications	52 (2.36)
Difficulty with at least one ADL	In total	378 (17.13)
	65 years and above	356 (20.46)
	75 years and above	266 (30.37)
Difficulty with at least one IADL	In total	789 (35.75)
	65 years and above	735 (42.24)
	75 years and above	502 (57.31)

### Assessment of the influence of factors significantly associated with disability

The logistic regression models included variables that significantly differentiated the studied population in terms of the occurrence of at least one problem with ADLs and IADLs. Due to the large number of missing answers (685 missing items), a variable for the income of the surveyed population was not included in the models.

The model of the effect of ADL factors was well adjusted to the data, as indicated by the results of the Hosmer-Lemeshow test (χ^2^_HL_ = 4.311, *p* = 0.828), and the pseudo R^2^ value was equal to 0.8559, indicating that the model correctly classified 85.59% of the cases. An important factor related to the occurrence of ADL limitations was the presence of barriers and obstacles in the respondent’s environment, including architectural, communication, social and other barriers. The presence of barriers and obstacles increased the risk of occurrence of at least one ADL limitation by almost four times (exactly 3.74 times) compared to that of people who did not report such barriers in their environment. Another important factor was engaging in daily physical activity that caused shortness of breath, sweating, and slight fatigue (e.g., doing housework, gardening, brisk walking, or participating in sports) for at least 30 min a day for a total of at least 150 min a week. People who did not engage in such activity had almost two and a half times greater odds (exactly 2.33 times) of having at least one ADL limitation. Moreover, maintaining social contacts was also a significant factor. People who did not maintain social contacts were twice as likely (exactly 2.04 times) to have at least one ADL limitation. Another important factor influencing ADL disability was maintaining good relations with relatives. People who did not maintain good relations with their family members were 1.5 times more likely to experience ADL disability than people with good relations with their family. Other important factors affecting ADL limitations were pain, age and the number of chronic diseases. With each successive level on the VAS scale, the odds of disability increased by 27%. In addition, with each subsequent year of participants’ age, the odds of disability increased by 8%, and with each subsequent chronic disorder, the odds increased by 7%.

The model of the effect of IADL factors was well suited to the data, as indicated by the results of the Hosmer-Lemeshow test (χ^2^_HL_ = 11,473, *p* value = 0.176) as well as a pseudo R^2^ value of 0.7748, indicating that the model correctly classified 77.48% of the cases. As in the ADL model, an important factor associated with the presence of IADL limitations was the existence of barriers and obstacles in the participant’s environment. The presence of barriers and obstacles increased the risk of at least one IADL limitation by three times (exactly 2.98 times) compared to that in people who did not have such barriers in their environment. Another important factor was participating in daily physical activity for a total of at least 150 min a week. Persons who did not perform such activity were almost one and a half times (exactly 1.36 times) more likely to live with at least one IADL limitation. Similarly, people who did not maintain social contacts had almost one and a half times (exactly 1.35) greater odds of having at least one IADL limitation. Other dominant factors affecting the occurrence of IADL limitations were pain, age and the number of chronic diseases. With each subsequent level of the VAS scale, the odds of disability increased by 27%. In addition, with each subsequent year of participants’ age, the odds of disability increased by 10%, and with each subsequent chronic illness, the odds increased by 4% (Table 3).

**Table 3:** Logistic regression models illustrating factors significantly associated with disability on at least one ADL and IADL of people aged 60 and more (*n* = 2207)

Variables		Difficulty with ADL			Difficulty with IADL	
	Odds Ratio	95% CI	*p* value	Odds Ratio	95% CI	*p* value
Age	1.08	(1.06–1.10)	< 0.001	1.10	(1.08–1.11)	< 0.001
Pain on the VAS scale	1.27	(1.20–1.34)	< 0.001	1.27	(1.22–1.33)	< 0.001
Number of diseases	1.07	(1.02–1.12)	< 0.001	1.04	(1.01–1.08)	0.023
Physical activity performed daily, with a minimum of 150 min per week (reference yes) no	2.33	(1.66–3.44)	< 0.001	1.36	(1.07–1.73)	0.013
Presence of barriers and obstacles (including architectural, communication, social and other barriers) in the environment of the respondent (reference no) yes	3.73	(2.51–5.54)	< 0.001	2.98	(2.32–3.83)	< 0.001
Maintenance of social contacts (reference yes) no	2.04	(1.41–2.97)	< 0.001	1.35	(1.06–1.72)	0.014
Maintenance of good relations with relatives (reference yes) no	1.50	(1.04–2.16)	0.003	–	–	–

### Specific pairs of factors significantly associated with disability

An assessment of the impact of pairs of factors that significantly differentiated the examined population was conducted in terms of the occurrence of at least one problem with ADLs or IADLs and they entered in to two logistic regression models presented above.

The incidence of disability varied widely depending on the specific pairs of factors, but the incidence significantly increased in each pair. The highest odds ratio of at least one ADL limitation was observed for the combination of barriers in the participant’s environment with multimorbidity (odds ratio (OR) 74.07), pain on the VAS scale ≥3 points (OR 50.93), a lack of at least 150 min of physical activity in a week (OR 44.51) or older age (OR 42.40). A very high odds ratio was also found for the combination of multimorbidity and a lack of social contacts (OR 47.50) (Table 4).

**Table 4:** Odds ratios (ORs) and 95% confidence intervals (CIs) testing the association of each different pair of factors significantly associated with difficulty with at least one ADL and IADL (*n* = 2207)

Variables		Difficulty with ADL			Difficulty with IADL	
	Odds Ratio	95% CI	*p* value	Odds Ratio	95% CI	*p* value
Age ≥ 75 years and pain on the VAS scale ≥3 point	24.41	(14.27–41.75)	< 0.001	19.47	(14.24–26.63)	< 0.001
Age ≥ 75 years and number of diseases ≥2	23.75	(10.45–53.99)	< 0.001	13.67	(9.1–20.53)	< 0.001
Age ≥ 75 years and lack of participation in physical activity performed daily for a minimum of 150 min per week	13.46	(8.08–22.44)	< 0.001	7.92	(5.89–10.66)	< 0.001
Age ≥ 75 years and presence of barriers and obstacles (including architectural, communication, social and other barriers) in the respondent’s environment	42.40	(23.95–75.06)	< 0.001	18.56	(14.06–24.51)	< 0.001
Age ≥ 75 years and a lack of social contacts	13.26	(9.36–18.8)	< 0.001	8.24	(6.4–10.61)	< 0.001
Age ≥ 75 years and a lack of good relations with relatives	12.91	(9.14–18.24)	< 0.001	8.17	(6.37–10.49)	< 0.001
Pain on the VAS scale ≥3 point and number of diseases ≥2	35.36	(11.26–111.00)	< 0.001	8.67	(5.92–12.69)	< 0.001
Pain on the VAS scale ≥3 point and lack of participation in physical activity performed daily for a minimum of 150 min per week	23.59	(9.64–57.74)	< 0.001	8.68	(5.91–12.76)	< 0.001
Pain on the VAS scale ≥3 point and presence of barriers and obstacles (including architectural, communication, social and other barriers) in the respondent’s environment	50.93	(23.86–108.71)	< 0.001	18.31	(13.47–24.88)	< 0.001
Pain on the VAS scale ≥3 point and a lack of social contacts	32.63	(17.97–59.24)	< 0.001	16.33	(11.75–22.71)	< 0.001
Pain on the VAS scale ≥3 point and a lack of good relations with relatives	33.29	(18.34–60.43)	< 0.001	16.28	(11.77–22.51)	< 0.001
Number of diseases ≥2 and lack of participation in physical activity performed daily for a minimum of 150 min per week	43.14	(6.01–309.70)	< 0.001	6.87	(3.91–12.07)	< 0.001
Number of diseases ≥2 and presence of barriers and obstacles (including architectural, communication, social and other barriers) in the respondent’s environment	74.07	(18.33–299.37)	< 0.001	17.68	(11.26–27.75)	< 0.001
Number of diseases ≥2 and a lack of social contacts	47.50	(15.08–149.62)	< 0.001	11.43	(7.46–17.51)	< 0.001
Number of diseases ≥2 and a lack of good relations with relatives	36.74	(13.54–99.68)	< 0.001	11.51	(7.56–17.52)	< 0.001
Lack of participation in physical activity performed daily for a minimum of 150 min per week and presence of barriers and obstacles (including architectural, communication, social and other barriers) in the respondent’s environment	44.51	(18.22–108.71)	< 0.001	8.02	(5.97–10.77)	< 0.001
Lack of participation in physical activity performed daily for a minimum of 150 min per week and a lack of social contacts	16.76	(9.60–29.28)	< 0.001	4.74	(3.58–6.27)	< 0.001
Lack of participation in physical activity performed daily for a minimum of 150 min per week and a lack of good relations with relatives	15.23	(9.00–25.78)	< 0.001	4.98	(3.76–6.59)	< 0.001
Presence of barriers and obstacles (including architectural, communication, social and other barriers) in the respondent’s environment and a lack of social contacts	19.37	(12.95–28.96)	< 0.001	8.73	(6.87–11.11)	< 0.001
Presence of barriers and obstacles (including architectural, communication, social and other barriers) in the respondent’s environment and a lack of good relations with relatives	20.41	(13.56–30.71)	< 0.001	9.20	(7.22–11.73)	< 0.001
A lack of social contacts and a lack of good relations with relatives	5.73	(4.42–7.44)	< 0.001	3.35	(2.76–4.08)	< 0.001

With regard to IADL disability, the highest odds ratio of at least one limitation was observed for the combination of pain on the VAS scale ≥3 points with older age (OR 19.47), a lack of social contacts (OR 16.33), or a lack of good relations with relatives (16.28). A high odds ratio of disability also was observed for the combination of barriers in the participant’s environment with older age (OR 18.57), pain (OR 18.31) or multimorbidity (OR 17.68) (Table 4).

## Discussion

In recent decades in Poland and worldwide, an extension of the average life expectancy and a significant increase in the number of older people in society have been observed [28]. The population of people over 60 is complex and heterogeneous in terms of health and functioning [29]. Therefore, while planning and designing health interventions in older persons, it is important to identify the factors that have the greatest impacts on the occurrence of disability in the performance of basic and complex activities of everyday life (ADLs and IADLs, respectively). It is also important to assess such disability in different regions of the world, especially those that are characterized by a high incidence of disability among older people.

Overall, in our study, we found a high prevalence of ADL and IADL limitations among older people over 60 living in southeastern Poland. Regarding the entire population discussed in our study, the percentage of people who reported at least one ADL limitation was 17.13%, and the percentage reporting at least one IADL limitation was 35.75%. In the population of people over 65, the percentage was even higher, at 20.46% for those reporting ADL limitations and 42.24% for those reporting IADL limitations. These percentages are higher than those observed in an Irish study, where 13% of people aged 65 and older had at least one ADL limitation, and 11% had at least one IADL limitation [9]. Chalise et al. also presented lower functional disability in ADL and IADL among Nepalese Newar elderly, aged 60 years and older. They showed that 8.7% had functional disability in at least one ADL item, and 29.2% reported functional disability in at least one IADL item. The percentage of people with functional disability increased in the group aged 65 and older and regarding ADL it was 12.8% and IADL 36.8% [30]. Problems with ADLs and IADLs significantly increased with age in the studied population. In people aged 75 and older, 30.37% had problems with ADLs, and 57.31% had problems with IADLs. Similar results were obtained by Wahrendorf et al., who compared the results of three large studies on the incidence and relationship of disability among older people (SHARE, ELSA and HRS), determining that ADL and IADL disability levels are the highest in Poland and the Czech Republic, especially among people aged 75 to 85 [8].

The percentage of people with ADL problems similar to the results of our study was found during the SAGE study carried out in six countries: China, Ghana, India, Mexico, the Russian Federation, and South Africa. It showed the occurrence of at least one problem in ADL in 27.7% of people aged 60–69 and up to 44.0% of those aged 70 and more [6]. A higher percentage of people aged 60 and more (mean = 71.8) with at least 1 problem in ADL was found by Germain et al. examining American older population (i.e. 36.2%) under the HRS (Health and Retirement Survey) program. However, they found a similar percentage of people with at least one IADL problem (37.1%) [31]. A higher incidence of at least one problem with ADL (53.5%) and IADL (66.8%) was found by Villarreal et al. in a group of people aged 65 and more living in Panama [32].

The strongest factor associated with ADL limitations in our study was the presence of barriers and obstacles in the respondent’s environment, including architectural, communication, social and other barriers. The presence of barriers and obstacles increased the risk of having at least one ADL limitation by almost four times and increased the risk of having at least one IADL limitation by three times compared to that of people who did not report such barriers in their environments. Environmental barriers, such as poor street conditions, high curbs, hills in a nearby environment, distance to service facilities, lack of benches, noise, heavy traffic, dangerous junctions, cyclists on road, presence of snow and ice, uncertainty due to other pedestrians, cars standing on the road, poor lighting and a lack of pedestrian zones, impair mobility [33] and reduce the sense of security [34]. Moreover, other important barriers are problems with access to transport and difficulties with access to health facilities [35]. Architectural barriers occurring at home are a frequent cause of falls and fractures; thus, they also increase fears of falling, thus limiting the activity of older people [36]. Consequently, barriers limit the activity of older people both at home and outside the home [37]. Limitation of activity leads to a decrease in functional condition and an increased risk of further ADL and IADL limitations [38]. The well understood living environment may actively influence the aging process. Elimination of barriers and implementation of facilitators, both at home and in the external environment, can significantly reduce the disability and increase the independence of older people [39].

In our study, we found that people who reported that they did not have good relations with their relatives were one and a half times more likely to have ADL disability. The inability to benefit from the help of other people creates serious barriers to the activity and participation of older people [40]. The possibility of having help in everyday functioning enables older people to continue to live in their own homes [41]. Family support allows older people to reduce the stress connected with chronic illnesses and reduced functional capacity [42].

Social contacts are another important factor. People who did not maintain social contacts were more than twice as likely to have at least one ADL limitation and had almost one and a half times greater odds of having at least one IADL limitation. The social participation of older people is important for their active aging. Social participation has a positive effect on the physical and mental health of older people, sustaining their performance of ADLs [43] and cognitive functions [44] and leading to a higher level of health-related quality of life [45]. This effect can be reinforced through participation in various organizations [43]. Previous studies have indicated that the social activity of older people is associated with a reduced risk of decline in motor function [46] and cognitive function [47], as well as disability in everyday life [48]. Poor social relationships increase the risk of mortality [49].

Another important factor is participation in daily physical activity that causes shortness of breath, sweating, and slight fatigue for at least 30 min a day for a total of at least 150 min a week. People who did not report such activity were almost two and a half times more likely to experience at least one ADL limitation. Physical activity is one of the most effective preventive and therapeutic factors reducing the risk of physical and mental disorders and affecting the maintenance of independence in everyday life [50]. One of the most important forms of physical activity for older people is walking because it not only allows the maintenance of motor functions but also fosters participation in the community [51].

In our study, we found that age was an important determinant of the functioning of older people. With each subsequent year of life, the odds of having problems with ADLs increased by 8%, and the odds of having problems with IADLs increased by 10%. The increase in the risk of ADL and IADL difficulties with age was also confirmed by other studies. Connolly et al. observed an approximately two- and a half-fold increase in the risk of functional ADL and IADL difficulties among Irish people in the 75–79 age group and a four-fold increase in risk in the 80 and older age group compared to that in the 65–69 group [9].

Moreover, in our study, we determined that with each subsequent chronic disease, the odds of having at least one problem with ADLs and IADLs increased (by 7 and 4%, respectively). Other studies have also confirmed that the level of disability increases with an increase in the number of chronic diseases [52, 53]. Marengoni et al. showed that the prevalence of disability was the lowest among people with cardiovascular diseases and the highest among people with mental and cerebrovascular diseases. In addition, the authors also demonstrated that combinations of diseases such as dementia, depression, cerebrovascular and musculoskeletal disorders were associated with the highest prevalence of disability [54].

Another important factor associated with problems with ADLs and IADLs was pain. The severity of pain caused a significant increase in the risk of disability, with each subsequent VAS point causing as much as a 27% increase in both ADL and IADL disability. This finding was confirmed by other studies. According to Connoll et al., there was a two-fold increase in the risk of ADL and IADL difficulties among older people who had pain compared to that of people who did not have such pain [9]. Moreover, Scudds et al. indicated that an increase in the intensity of pain also increased the risk of disability; in the presence of moderate pain, the OR was 1.54, while in the presence of severe and extreme pain, the OR was 4.32 [55]. Moreover, Andrews et al. noted that pain is strongly associated with the disability of older people and causes disability in a short time. Therefore, the assessment of pain in older persons is very important because it allows health care workers to identify people who have a potentially reversible cause of functional limitations and disabilities, especially in the early stages of the development of symptoms [56].

Regarding our study, we found that the occurrence of pairs of factors that we repeatedly analyzed increased the odds of limitations in the functioning of older people. In particular, the combination of the presence of barriers and obstacles in the living environment of an older person with multimorbidity, pain, or older age affected the likelihood of experiencing at least on ADL or IADL limitation. Moreover, in the case of ADL disability, the combination of barriers in the environment with a low level of physical activity was important. In the presence of these combinations, the odds of at least one ADL problem in older people increased several dozen times compared to that of people who did not report such combinations of factors. The majority of older adults in Poland want to stay in their own homes in the later years of their life, but due to disability, they are often forced to make decisions about institutionalization. Roy et al. showed that 25% of factors influencing older people’s decisions about changing their places of residence were related to barriers in the house and its surroundings [57]. Most dwellings of older people who suffer from chronic diseases are not adapted to their functional status and make everyday activities troublesome [58]. It is difficult to compare the results of our study with those of others because there are limited data assessing older people’s places of residence in terms of barriers or facilitators.

We have shown that the odds of ADL disability also increase significantly with a pair of factors such as multimorbidity and lack of social contacts. Older people with chronic diseases seem to be less involved in social life and to experience more barriers that prevent them from active participation. Despite the growing importance of this subject matter, studies assessing the level of participation in social life among older people with chronic diseases are rare [59]. Active participation and involvement in social life are very important for older people and positively influence their psychophysical condition. Therefore, the challenge for the government is to facilitate older people’s social participation despite their health limitations.

In the case of IADL disability, a high OR of at least one limitation was observed when combining a higher level of pain and older age, as well as pain and a lack of social contacts or a lack of good relations with relatives. Pain is a frequent factor hindering the movement of older people over long distances and thus their ability to manage many complex activities located away from home [9]. In addition, a lack of relationships with relatives or social contacts increases the difficulty of receiving help in performing various complex activities, negatively affecting the psychophysical conditions of older adults in Poland [60]. Micheli et al. found that respondents with worse family relations had a higher risk of functional limitations [61]. It is important to develop a network of contacts and build social relations among older persons to arouse their motivation to be active and participate in neighborly assistance [62].

Our results confirm the range of problems that older people encounter in Poland and show how urgent and necessary it is to modify the support system in our country. Difficult access to medical, rehabilitation and social care is associated with a long waiting time for these services. The increase in the number of single-person households and the breakdown of multigenerational households result in loneliness and a lack of support for older people. Poor housing conditions often make it difficult to take care of older adults. These are the most urgent problems of older persons in Poland [63]. In addition, the low participation of older people in active social life and the implementation of the idea of “active aging” in Poland for only several years means that the oldest people are now largely excluded from active life in society [64].

The identification of the factors or groups of factors most strongly associated with the occurrence of disability is important in the context of prevention and planning care for older people. It has been shown that medical expenses in the older adult population are more connected with disability than longevity [65]. New strategies for disability prevention should be focused on the presence of a combination of risk factors.

### Limitations

This study has some limitations. First, the cross-sectional nature of this study does not allow the researchers to make strict cause effect interpretations of the associations between ADL and IADL disability and its determinants. A longitudinal study is recommended to establish such associations. Second, the population of older people under institutional care was excluded from the study, and therefore, the prevalence of disability may have been completely underestimated.

## Conclusions

In summary, our study revealed a high prevalence of ADL and IADL disability in older people living in southeastern Poland. Environmental barriers, a lack of social contacts, multimorbidity and increased pain are the factors with the strongest influence in increasing the odds of ADL and IADL disability in the studied population of older people. In the case of problems with ADLs, a very strong factor is also a lack of good relations with relatives and the possibility of receiving their support in everyday life. Pairs of factors, especially those including environmental barriers, significantly increase the odds of limitations to the functioning of older people. Practitioners must be aware of these links and take into account the environmental factors and social and family relationships of patients to develop individual strategies for disability prevention. Researchers should fill the gap in the literature by considering the assessment of environmental barriers and facilitators and their impacts on the prevalence of disability among older people.

## Data Availability

All data used in this study was stored at https://repozytorium.ur.edu.pl/handle/item/4921.

## Abbreviations

ADL: Activities of daily living
AMTS: Abbreviated mental test score
AMTS: Abbreviated mental test score
BMI: Body mass index
IADL: Instrumental activities of daily living
ICF: International classification of functioning, disability and health
OR: Odds ratio
WHO: World Health Organization
